# Nutritional Psychiatry: How Diet Affects Brain through Gut Microbiota

**DOI:** 10.3390/nu13041282

**Published:** 2021-04-14

**Authors:** Giuseppe Grosso

**Affiliations:** Department of Biomedical and Biotechnological Sciences, University of Catania, 95123 Catania, Italy; giuseppe.grosso@unict.it; Tel.: +39-0954-781-187

**Keywords:** diet, dietary factors, lifestyle, gut microbiota, nutritional psychiatry, mental health, sleep, cognitive, mood, anxiety

## Abstract

Nutritional sciences have been recognized as being of paramount importance for the prevention of non-communicable diseases. Among others, mental health disorders have been hypothesized to be influenced by dietary risk through a variety of molecular mechanisms. The improvements in the technology and implementation of-omics sciences in terms of nutrition have created the possibility of studying the relation between diet, gut microbiota and mental health. The gut–brain–axis represents the core rationale setting the stage for a relatively new discipline of study defined as “nutritional psychiatry”. Research on this matter will help to better understand the relation between food and mood, sleep quality, cognition, and mental health in general.

Mental health disorders have risen as common morbidities of this century [1]. They are characterized by a number of clinical conditions, among which, stress, anxiety, and depression are the most common, which could evolve and mutate into far more dangerous illnesses, including psychosis and schizophrenia [2]. In 2017, it was estimated that mental disorders accounted for about 14% of worldwide years lived with disability (depression alone accounted for over 50 million and anxiety disorders about half of that) [3]. The reasons for such a rise in the incidence of mental health disorders is unknown: from an epidemiological point of view, a better knowledge of these conditions allowed a more timely recognition and consequently, a higher rate of diagnosis. However, an increased incidence of such illnesses has been recognized, and a common hypothesis relies on the modern lifestyle and the “stressogenic” environment we live in [4,5]. In fact, lifestyles have greatly changed over the last 50 years: urbanization and the technological improvements up to the so-called “information revolution”, the modern lifestyle characterized by longer days (due to night lights), the long screen-hours, accompanied by the pressure from a competitive society may finally result in a mismatch with the human genetic heritage, largely unchanged from our ancestors [6]. The resulting trends over the future projections of incidence of mental diseases are thus alarming, being anticipated that by 2030 mental health diseases will be the leading cause of disease burden globally [7].

While pharmacological therapies have been of primary use and utility to cure mental disorders, behavioral interventions have caught on in recent decades as support for conventional therapy. However, nearly no progress has been made regarding the prevention of such conditions, as no univocal risk factor has been identified. With the discovery of inflammation playing a role in several central nervous system diseases [8], an intriguing hypothesis has been postulated not long ago, suggesting that dietary factors may play a role in mental health diseases [9]. From a mechanistic point of view, a potential direct anti-inflammatory effect (i.e., omega-3 polyunsaturated fatty acids), antioxidant action (i.e., polyphenols able to pass the blood–barrier membrane, such as anthocyanins, etc.), or functional modulation (i.e., group B vitamins, glycine, L-ornithine, tryptophan amino acids, etc.), may provide the rationale for the potential effects of diet on mental health [10]. This evidence has set the stage for a new discipline of study defined as “nutritional psychiatry” [11].

Today, nutritional support as supplements or dietary interventions characterize adjuvant therapy against depression, anxiety, stress, and cognitive decline [12,13,14,15,16]. Moreover, a great deal of studies have been conducted relating dietary variables and the prevention of mental health disorders [17,18,19]. Together with the aforementioned mechanisms, current evidence suggests that the rich innervation of the gastrointestinal system might deliver impulses and signals to the brain in addition to receiving them [20]. In this context, the gut microbiota may play an important role in the integrity and proper functionality of the human gut: certain dietary factors may affect the intestinal microbiome, resulting in the alteration of nutrient absorption, weakening of the intestinal barrier against toxins and bacteria per se, the determination of chronic inflammation, and subsequently, the activation of neural pathways that directly affect the functionality of the central nervous system [21]. Alterations of gut microbiota have been demonstrated in association with changes in food intake or adherence to an entire dietary pattern (in an healthy or unhealthy direction) [22]. Recent evidence also suggests that circadian rhythms and feeding time (i.e., intermittent fasting or time-restricted feeding) may also play a role in the gut microbiota profile, with consequent potential effects on systemic inflammation and mental health outcomes.

The Special Issue “Recent Advances in Nutritional Psychiatry” provided new and interesting insights on this matter including cognitive status, depression, and sleep quality. The study of Fisicaro et al. [23] showed that mocha (stove) coffee consumption may be associated with improved cognitive and mood status. Currenti et al. [24] also provided the first evidence that not only diet quality features, but also time of eating may play a role on cognitive status: specifically, individuals having their eating time restricted to 10 h were less likely to have cognitive impairment in a cohort of southern Italian older adults. A laboratory study explored the hypothesis that the content in anthocyanins of isogenic wheat lines may be determinant to exert positive effects on neurodegenerative disorders [25]. Concerning sleep quality, two studies have shown an association of higher adherence to the Mediterranean diet during pregnancy [26] and food security with sleep quality [27], respectively; a third study from our group provided the first evidence of a potential role played by dietary polyphenol content in sleep quality [28]. Diet quality has also been related to depression in two studies: Cebrino et al. [29] reported that non-depressive individuals had a higher diet quality than depressive ones in a nationwide cross-sectional study conducted in Spain; in the study of Marozoff et al. [30], the authors showed that increasing Healthy Eating Index-Canada scores were associated with fewer physician visits for depression in a prospective investigation of adults living in Alberta (Canada).

The review of Janda et al. [31] summarized the evidence from clinical trials of the therapeutic effects of Passiflora incarnata in neuropsychiatric disorders showing potential for its use against anxiety symptoms with no adverse effects to mention. Finally, the review of Wlodarczyk et al. [32] points out that the ketogenic diet might be used as an add on to common psychotherapy and pharmacology for anxiety disorders.

The growing number of studies related to nutritional psychiatry corroborates the need for a better understanding of the relation between dietary factors and mental health disorders. Future studies should fill the gap between the epidemiological and clinical evidence of the prevention of mental health disorders through dietary factors by investigating mechanistic features related to gut microbiota and its interaction with the central nervous system.

## Data Availability

Not applicable.
